# Association of Subclinical Inflammation Markers with Primary Hypertension in Children—A Systematic Review with Meta-Analysis

**DOI:** 10.3390/jcm14072319

**Published:** 2025-03-28

**Authors:** Katarzyna Dziedzic-Jankowska, Maciej Kołodziej, Piotr Skrzypczyk

**Affiliations:** 1 Department of Pediatrics and Nephrology, Medical University of Warsaw, 02-091 Warsaw, Poland; katarzyna_dziedzic11@wp.pl; 2 Department of Pediatrics, Medical University of Warsaw, 02-091 Warsaw, Poland; maciej.kolodziej@wum.edu.pl

**Keywords:** meta-analysis, primary hypertension, children, subclinical inflammation, biomarkers

## Abstract

**Background/Objectives**: This systematic review and meta-analysis aimed to determine whether there is an association between low-grade inflammation markers and primary hypertension (PH) in children. **Methods**: The MEDLINE, EMBASE, and Cochrane databases were searched up to March 2025 for cohort, cross-sectional, and case–control studies; additional references were obtained from reviewed articles. The studies needed to investigate an association between any inflammation markers and PH. Participants of the study were children (<18 years old) with PH and healthy controls. This meta-analysis included 13 studies published between 2005 and 2024, enrolling 1306 patients (745 with PH and 561 healthy controls). The data were analyzed using Review Manager. Pooled mean difference (MD) with a 95% confidence interval (95% CI) was used to assess the differences in inflammation markers. **Results**: There was a significant difference between hypertensive and control groups in high-sensitivity C-reactive protein (hs-RCP) concentration (mean difference (MD): 0.07 95%CI (0.04, 0.09)), intercellular adhesion molecule 1 (ICAM-1) (MD: 85.28 95%CI: (50.57–119.99)), vascular cell adhesion molecule 1 (VCAM-1) (MD: 259.78 95%CI: (22.65–496.91)), neutrophil count (MD: 0.90 95%CI (0.66–1.14)), monocyte count (MD: 0.08 95CI%: (0.04–0.11)), platelet count (MD: 20.24 95CI%: (4.27–36.21)), neutrophil-to-lymphocyte ratio (MD: 0.48 95%CI: (0.34–0.62)), and lymphocyte-to-monocyte ratio (MD: −0.52 95%CI: (−1.02–−0.02)). There was no difference in terms of interleukin 6 (IL-6), lymphocyte count, mean platelet volume (MPV), or platelet-to-lymphocyte (PLR) ratio. **Conclusions**: Some easily accessible markers of low-grade inflammation might be used as an additional tool for diagnosis and screening for hypertension in children. These results should be validated in large and well-conducted studies.

## 1. Introduction

Arterial hypertension (AH) is a global public health problem and one of the most common chronic diseases worldwide [1]. Also, according to a recent systematic literature review, its prevalence in developmental age is estimated at 4.0% [2]. Primary hypertension (PH) is a dominant form of arterial hypertension in adults. In contrast, in children and adolescents, secondary causes are found in approximately half of the individuals with elevated blood pressure [3]. PH is by far the most common form of AH in adolescents [4]. Of note, the prevalence of PH in children and teenagers is rising in many countries as a consequence of the increasing prevalence of obesity, a sedentary lifestyle, and an unhealthy diet rich in fat, simple sugars, and primarily excessive salt intake.

Primary hypertension is not only a cardiovascular disease but also a multisystem disorder with complex, only partially uncovered, pathogenesis. Genetic and environmental factors activate numerous systems, including the sympathetic, renin–angiotensin–aldosterone, and immune system, which lead to blood pressure elevation and the development of hypertension-mediated organ damage (HMOD) [5].

The role of immune system activation in the pathogenesis of primary hypertension has been extensively studied for the last 20 years [6]. Low-grade (subclinical) inflammation is involved in the pathogenesis of endothelial dysfunction, leading to structural and functional changes in the endothelium, often in the early stages of hypertension. Also, the immune system is considered one of the factors responsible for salt sensitivity, exacerbation of arterial hypertension, and formation of HMOD [7].

There are a lot of studies concerning adults that show that acute phase proteins, including high-sensitivity C-reactive protein (hs-CRP), interleukins (IL)-IL-6, IL-18, and other inflammatory markers, e.g., complete blood count-derived indicators such as neutrophil-to-lymphocyte ratio (NLR) or platelet-to-lymphocyte ratio (PLR), are early markers of the immune system activation in PH and have been associated with different cardiovascular diseases and their complications [8,9,10,11,12,13].

Not much is known about the relationship between subclinical inflammation and primary hypertension in the pediatric population or about the usefulness of the markers mentioned above in young hypertensive patients. Single, small, case–control pediatric studies preclude drawing conclusions on this topic [14].

Thus, we conducted a systematic review to summarize existing evidence on the association between subclinical inflammation and PH in pediatric patients.

## 2. Materials and Methods

This systematic review of observational studies investigated the association between subclinical inflammation markers and PH in pediatric patients. Our work was performed following the recommendations of the 2020 Preferred Reporting Items for Systematic Review and Meta-analysis (PRISMA) guidelines [15] and using the Cochrane Handbook for Systematic Reviews guidelines. The study protocol was registered in the International Prospective Register of Systematic Reviews at the National Institute for Health Research and Centre for Reviews and Dissemination of the University of York (PROSPERO registration number: CRD42022377144); no amendments were made to the protocol since its registration.

### 2.1. Criteria for Considering Studies for This Review

We included observational studies: cohort, cross-sectional, and case–control studies. The included studies needed to investigate an association between any inflammation markers and PH defined by the authors in pediatric patients.

### 2.2. Search Methods for Identification of Studies

The Cochrane Central Register of Controlled Trials (CENTRAL), Pubmed, and EMBASE databases were searched for relevant studies up to March 2023, and then the search was updated until March 2025. Three reviewers (KD, MK, PS) independently carried out the search without any language restrictions. For the search strategy, please see Table S1.

### 2.3. Study Selection and Analysis

The titles of identified studies and the abstracts of relevant articles were screened. Full texts were retrieved for each study potentially relevant for inclusion. The articles’ eligibility and the whole study identification and screening process were assessed independently by three authors (KD, MK, and PS). In case of disagreement, it was resolved by the discussion process.

### 2.4. Data Extraction and Management

Using a standard data extraction form, three reviewers (KD, MK, and PS) independently extracted information from each included study. The extracted data included author, year, study design, country, number of participants, analyzed markers of subclinical inflammation, preliminary hypertension definition, and results.

### 2.5. Risk of Bias Assessment

The risk of bias was assessed using the Newcastle–Ottawa Scale (NOS) [16]. Three reviewers (KD, MK, and PS) independently performed the assessment, with disagreements resolved by discussion. The NOS tool uses a “star system” in which a study is evaluated based on the following criteria of study group selection (4 items, equal to 4 stars), comparability of groups (2 items), and ascertainment of either the exposure or outcome (3 items). Overall, the Newcastle–Ottawa Scale scores vary between 0 and 9 (9 is the highest level of quality).

The literature analysis involved independent experts to minimize the risk of bias further. An independent recognized expert from the Systematic Reviews Unit, Jagiellonian University Medical College, Krakow, Poland (MK) prepared the search strategy. In addition, three independent researchers involved in arterial hypertension, pediatrics, and research methodology from the Department of Pediatrics (JŁ), Department of Pediatrics and Nephrology (BL), and Department of Internal Medicine, Hypertension and Vascular Diseases, Medical University of Warsaw (PS), Warsaw, Poland, were asked to analyze the selection of the papers included in the meta-analysis. The experts had access to the search strategy and had the complete list of analyzed manuscripts and full texts of the documents included in the meta-analysis. All three experts approved the selection of the papers in complete agreement.

### 2.6. Data Synthesis and Analysis

The data were analyzed using Review Manager (RevMan Version 5.4.1 Copenhagen: The Nordic Cochrane Centre, The Cochrane Collaboration, London, UK, 2014). Pooled mean difference (MD) with a 95% confidence interval (95% CI) was used to assess the differences in inflammation markers levels between the patients with HP and the control groups. Heterogeneity across the included studies was calculated using I^2^ statistics and the Q test. The I^2^ values showed serious (I^2^ = 75–100%), high (I^2^ = 50–74.9%), moderate (I^2^ = 25–49.9%), low (I^2^ = 0.1–24.9%), and no (I2 = 0) heterogeneity. For all analyses, we used the random-effect model. The missing means and standard deviations (SDs) were estimated using the formula recommended by Hozo et al. [17].

## 3. Results

**Figure 1:**
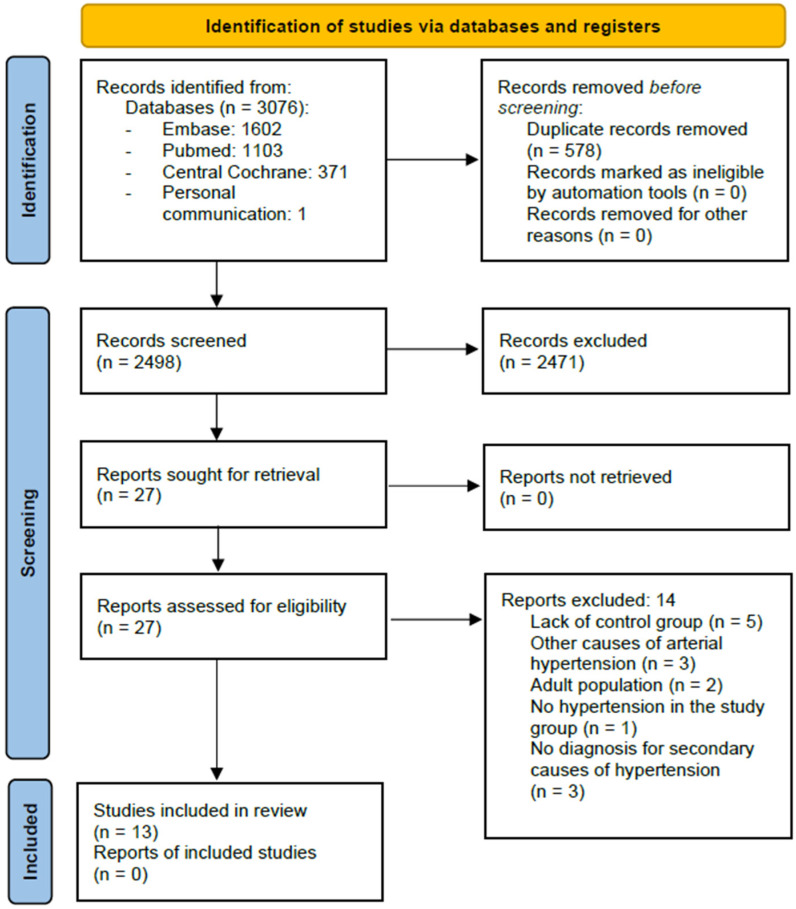
Study flow diagram according to [15].

For a flow diagram documenting the identification process for eligible trials, please see Figure 1. Characteristics of the included RCTs are presented in Table 1, and characteristics of the excluded trials are shown in Table S2.

We included 13 studies published between 2005 and 2024, enrolling 1306 patients (745 with PH and 561 healthy controls) [14,18,19,20,21,22,23,24,25,26,27,28]. The sample size ranged from 20 to 143 participants. Twelve studies were conducted in Poland and one in China.

**Table 1:** Characteristics of the included studies.

Author, Publication Year (Ref.)	Study Design	Country	Population (n)	Markers	Preliminary Hypertension Definition	Results
Dziedzic-Jankowska et al. [29]	Case–control	Poland	Untreated children with PH n = 56, CG n = 30	hs-CRP (mg/L), IL-18 (pg/mL), neutrophils (10^9^/L), lymphocytes (10^9^/L), monocytes (10^9^/L), platelets (10^9^/L), MPV (fL), NLR, PLR, LMR, MNR, PMPVR	Arterial hypertension was diagnosed according to the ESH 2016 guidelines and confirmed by ABPM	hs-CRP (2.9 (1.5–7.3) vs. 0.9 (0.5–1.9)); neutrophil count (3.89 ± 1.44 vs. 2.63 ± 0.96; *p* = 0.001), monocyte count (0.53 (0.45–0.65) vs. 0.44 (0.34–0.53); *p* = 0.026) were significantly higher in children with PH
Gackowska et al., 2020 [14]	Case–control	Poland	Untreated children with PH n = 33, CG n = 35	hs-CRP (mg/dL)	Arterial hypertension was diagnosed according to the ESH 2016 and American 2004 guidelines and confirmed by ABPM	hs-CRP concentration was significantly higher in children with PH (0.7 ± 0.7 vs. 0.4 ± 0.1; *p* = 0.02)
Garanty-Bogacka et al., 2005 [18]	Case–control	Poland	Children with PH n = 50, CG n = 143 No data on anti-hypertensive treatment	hs-CRP (mg/L), IL-6 (pg/mL), ICAM-1 (ng/mL), VCAM-1 (ng/mL)	Arterial hypertension was recognized based on ABPM when 24 h systolic and/or diastolic BP values exceed 95 th percentile for sex and height	hs-CRP (1.7 ± 0.9 vs. 0.9 ± 0.4; *p* < 0.001), IL-6 (2.1 (0.7–14.8) vs. 1.2 (0.1–3.6); *p* < 0.001), ICAM-1 (331.2 ± 138.3 vs. 230.9 ± 109.3; *p* < 0.001) and VCAM-1 (1258.1 ± 368.3 vs. 872 ± 439.1; *p* < 0.001) were significantly higher in children with PH
Głowińska- Olszewska et al., 2007 [19]	Case–control	Poland	Children with PH n = 31 CG n = 26 No data on anti-hypertensive treatment	ICAM-1 (ng/mL), VCAM-1 (ng/mL), E-selectin (ng/mL)	Arterial hypertension was diagnosed when at least 30% of the 24 h ABPM recordings exceeded the 95th percentile, matched for age and gender	ICAM-1 (319.6 ± 137 vs. 255.2 ± 43; *p* = 0.02), VCAM-1 (540.7 ± 209 vs. 396.8 ± 57; *p* = 0.02) and E-selectin (87.4 ± 28 vs. 65.4 ± 22; *p* = 0.001) were significantly higher in children with PH
Hou et al., 2021 [20]	Case–control	China	Untreated children with PH n = 65, CG n = 54	hs-CRP (mg/dL), WBC (10^9^/L), neutrophils (10^9^/L), lymphocytes (10^9^/L), monocytes (10^9^/L), platelets (10^9^/L), NLR, PLR, LMR	Arterial hypertension was defined as systolic and/or diastolic pressure ≥95 th percentile for sex, age, and height according to the reference values of the Chinese Child Blood Pressure References Collaborative Group	hs-CRP (2.22 ± 5.03 vs. 0.32 ± 0.42; *p* =0.004), WBC (7.65 ± 2.27 vs. 6.70 ± 1.71; *p* = 0.017), neutrophil count (4.62 ± 1.72 vs. 3.77 ± 1.27; *p* = 0.003) and NLR (2.18 ± 1.12 vs.1.68 ± 0.75; *p* = 0.005) were significantly higher in children with PH
Kołakowska et al., 2018 [21]	Case–control	Poland	Untreated children with PH n = 58, CG n = 30	hs-CRP (mg/L)	Arterial hypertension was confirmed by ABPM (24 h SBP or DBP >95th percentile for gender and height and SBP or DBP load > 25%)	hs-CRP (1.05 (0.54–1.33) vs. 0.17 (0.16–0.20), *p* < 0.01) was significantly higher in children with PH
Litwin et al., 2010 [22]	Case–control	Poland	Untreated children with PH n = 44, CG n = 30	hs-CRP (mg/L), MCP-1 (pg/mL), MIP-1β (pg/mL), MIP-1α (pg/mL), TNF-α (pg/mL), angiogenin (ng/mL), IL-6 (pg/mL), RANTES (ng/mL)	Normal office blood pressure values were taken from the Updated 4th Task Force Report. Diagnosis of arterial hypertension was confirmed by ABPM—SBP and/or DBP ≥ 95th percentile.	hs-CRP (1.2 ± 1.1 vs. 0.3 ± 0.2; *p* = 0.0001), MIP-1β (117.9 ± 140.6 vs. 58.3 ± 21.9; *p* = 0.04), and RANTES (19.7 ± 25.8 vs.10.7 ± 10.6; *p* = 0.04) were significantly higher in children with PH
Musiał et al., 2022 [23]	Case–control	Poland	Untreated children with PH n = 70, CG n = 20	hs-CRP (mg/L), neutrophils (10^3^/µL), platelets (10^3^/µL), lymphocytes (10^3^/µL), monocytes (10^3^/µL), NLR, PLR, LMR	Arterial hypertension was diagnosed according to 2016 ESH guidelines, based on three independent oscillometric office blood pressure measurements showing values > 95th percentile for age, sex, and height	NLR (2.0 ± 1.0 vs.1.5 ± 0.5; *p* < 0.05), PLR (135.6 ± 43.2 vs.121.4 ± 45.6; *p* < 0.05) were significantly higher and LMR (4.0 ± 1.4 vs. 4.9 ± 1.5; *p* < 0.05) was significantly lower in children with PH
Skrzypczyk et al., 2018 [24]	Case–control	Poland	Untreated children with PH n = 54, CG n = 20	Neutrophils (10^3^/µL), lymphocytes (10^3^/µL), platelets (10^3^/µL), NLR, PLR, MPV (fL)	Arterial hypertension was defined as systolic and/or diastolic pressure ≥ 95th percentile for sex, age, and height during 24 h according to AHA guidelines	There were no significant differences in evaluated inflammatory markers between children with PH and the control group
Skrzypczyk et al., 2021 [25]	Case–control	Poland	Children with PH n = 119 (55/119 on pharmacological treatment), CG n = 45	neutrophils (10^3^/µL), lymphocytes (10^3^/µL), platelets (10^3^/µL), NLR, PLR, MPV (fL)	Arterial hypertension was diagnosed according to Polish 2019 guidelines	Neutrophil count (3.9 ± 1.7 vs. 3.0 ± 1.0; *p* < 0.001), platelet count (271.9 ± 62.3 vs. 250.3 ± 60.3; *p* = 0.047), NLR (1.9 ± 1.5 vs. 1.3 ± 0.4; *p* = 0.01) and PLR (131.4 ± 41.9 vs. 114.7 ± 37.6; *p* = 0.02) were significantly higher in children with PH
Skrzypczyk et al., 2022 [26]	Case–control	Poland	Untreated children with PH n = 28, CG n = 25	NLR, PLR, MPV (fl)	Arterial hypertension was diagnosed according to Polish 2019 guidelines	No significant differences in evaluated inflammatory markers between children with PH and the control group
Trojanek et al., 2019 [27]	Case–control	Poland	Untreated children with PH n = 80, CG n = 78	hs-CRP (mg/L)	Arterial hypertension was diagnosed according to the 2016 ESH guidelines and confirmed by 24 h ambulatory blood pressure monitoring	hs-CRP (0.32 ± 0.18 vs. 0.12 ± 0.13; *p* = 0.0001) was significantly higher in children with PH
Wasilewska et al., 2010 [28]	Case–control	Poland	Untreated children with PH n = 57, CG n = 25	hs-CRP (mg/L), platelets (10^3^/µL), MPV (fL)	Arterial hypertension was diagnosed when SBP or DBP was ≥95th percentile (according to Polish normative values) and confirmed by 24 h ABPM	hs-CRP (0.66 (0.76–1.19) vs. 0.17 (0.14–0.31); *p* < 0.01), platelet count (284.5 (265.09–302.98) vs. 245 (232.75–268.2); *p* < 0.05) and MPV (11.3 (10.94–11.37) vs.10.3 (10.09–10.77); *p* < 0.01) were significantly higher in children with PH

ABPM—ambulatory blood pressure monitoring, AHA—American Heart Association, CG—control group, DBP—diastolic blood pressure, ESH—European Society of Hypertension, hs-CRP—high sensitivity c-reactive protein, ICAM-1—intercellular adhesion molecule 1, IL-6—interleukin 6, IL-18—interleukin 18, LMR—lymphocyte-to-monocyte ratio, MCP-1—monocyte chemoattractant protein 1, MIP-1α—macrophage inflammatory protein 1α, MIP-1β—macrophage inflammatory protein 1β, MNR—monocyte-to-neutrophil ratio, MPV—mean platelet volume, NLR—neutrophil-to-lymphocyte ratio, PH—primary hypertension, PLR—platelet-to-lymphocyte ratio, PMPVR—platelet-to-mean platelet volume ratio, RANTES—regulated on activation, normally T-expressed, SBP—systolic blood pressure, TNF-α—tumor necrosis factor α, VCAM-1—vascular cell adhesion molecule 1; WBC—white blood cells.

### 3.1. Risk of Bias of Included Studies

Overall, the included studies were at moderate risk of bias. Table 2 shows the risk of bias summary and judgments about each risk of bias domain per included study. A high risk of bias was observed in two domains concerning the selection and definition of control groups. In two studies, the control group consisted of hospital patients [21,28]. Also, seven studies did not describe the source of the control group [18,22,23,24,25,26,29]. Ten studies showed no adjustments for body mass index [14,20,21,22,23,24,25,26,28,29].

### 3.2. High-Sensitivity C-Reactive Protein

**Figure 2:**
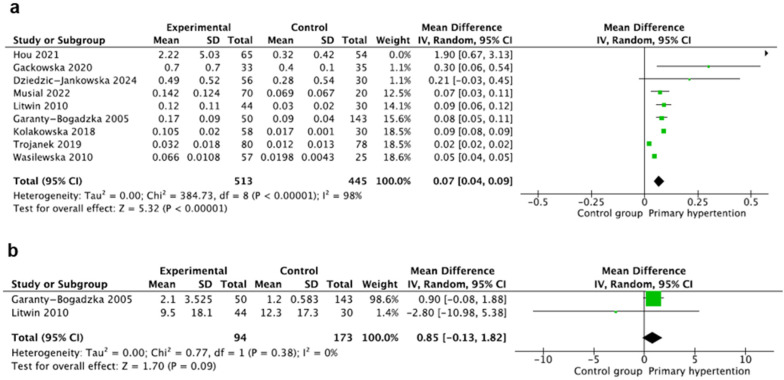
Meta-analysis of the mean value of high-sensitivity C-reactive protein (hs-CRP) (**a**) and interleukin 6 (IL-6) (**b**) [14,18,20,21,22,23,27,28,29].

This marker was evaluated in nine studies [14,18,20,21,22,23,27,28,29] including 958 participants, with 513 children with PH and 445 controls. Hs-CRP was measured using the nephelometric method [21,23,28], the immunoturbidimetric method [14,18,22], and the enzyme-linked immunoassay (ELISA) method [29]. The method was not specified in the studies by Trojanek and Hou. The unit of hs-CRP was (mg/L) in all studies except for the studies by Hou and Gackowska (both (mg/dL)). We recalculated all hs-CRP values to mg/dL. Hs-CRP was reported as mean ± SD except for the studies by Wasilewska and Kołakowska, where the authors provided only medians, and mean and SD values were calculated using the Hozo method [17]. The pooled results of hs-CRP (mg/dL) showed a significant increase in hs-CRP concentrations in the PH group, where MD: 0.07 95%CI: (0.04–0.09). There was significant heterogeneity (I^2^ = 98%) (Figure 2a).

### 3.3. Interleukin 6

Interleukin 6 (IL-6) was evaluated in two studies including 267 participants, with 94 children with PH and 173 children in the control group [18,22]. IL-6 was assessed using the ELISA method [18] and multiplexed bead-based immunoassay method [22], and the unit was (pg/mL) in both studies. IL-6 was reported as mean ± standard deviation in the study by Litwin [22]. In the second study, the authors provided medians, and mean and SD values were calculated with the Hozo method [17]. The mean value of IL-6 (pg/mL) did not differ significantly between the groups, where MD: 0.85 95%CI: (−0.13–1.82) and I^2^ = 0% (Figure 2b).

### 3.4. Intercellular Adhesion Molecule 1

**Figure 3:**
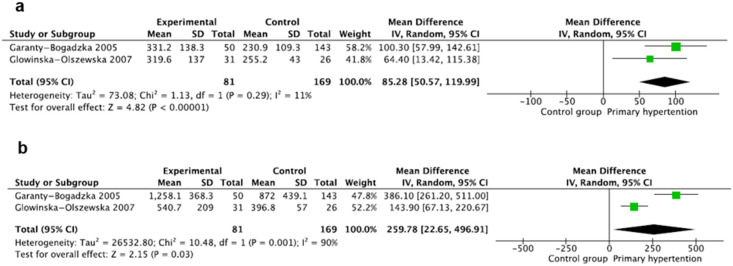
Meta-analysis of mean values of intercellular adhesion molecule 1 (ICAM-1) (**a**) and vascular cell adhesion molecule 1 (VCAM-1) (**b**) [18,19].

Intercellular adhesion molecule 1 (ICAM-1) was evaluated in two studies [18,19] including 250 participants, with 81 children with PH and 169 children in the control group. ICAM-1 was evaluated using the ELISA method, reported in (ng/mL), and expressed as mean ± SD in both studies. The pooled results showed that the mean value of ICAM-1 (ng/mL) was significantly higher in the hypertension group, where MD: 85.28 95%CI: (50.57–119.99); there was no significant heterogeneity between the studies, where I^2^ = 11% (Figure 3a).

### 3.5. Vascular Cell Adhesion Molecule 1

Vascular cell adhesion molecule 1 (VCAM-1) was also evaluated in two studies [18,19] including 250 participants, with 81 children with PH and 169 children in the control group. VCAM-1 was evaluated using the ELISA method, reported in (ng/mL), and expressed as mean ± SD in both studies. The pooled results showed that the mean value of VCAM-1 was significantly higher in the hypertensive children, where MD: 259.78 95%CI: (22.65–496.91); there was significant heterogeneity between the studies, where I^2^ = 90% (Figure 3b).

### 3.6. Neutrophils

**Figure 4:**
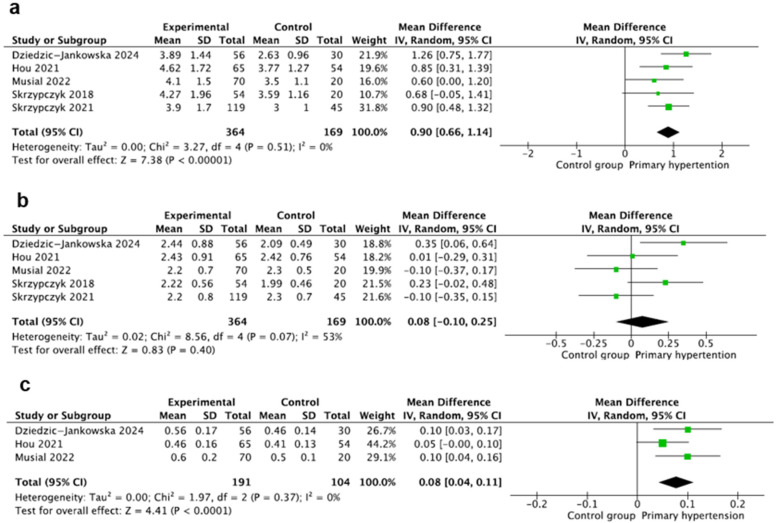
Meta-analysis of mean values of neutrophils (**a**), lymphocytes (**b**), and monocytes (**c**). [20,23,24,25,29].

This marker was evaluated in five studies [20,23,24,25,29] including 533 participants, with 364 children with PH and 169 controls. Neutrophil counts were calculated using standard hematologic analyzers, expressed in (1000/µL), and reported as mean ± SD in all studies. The pooled mean neutrophil count was significantly higher in the hypertension group (MD: 0.90, 95% CI: (0.66–1.14), I2 = 0%) (Figure 4a).

### 3.7. Lymphocytes

This marker was also evaluated in five studies [20,23,24,25,29] including 533 participants, with 364 children with PH and 169 children in the control group. Lymphocyte counts were calculated using standard hematologic analyzers, expressed in (1000/µL), and reported as mean ± SD in all studies, except for the study by Jankowska-Dziedzic (medians and interquartile ranges (IQRs)) [29]. Pooled analysis of mean lymphocyte count showed no differences between the groups, where MD: 0.08 95CI%: (−0.10–0.25). The heterogeneity of the studies was not significant, where I^2^ = 53% (Figure 4b).

### 3.8. Monocytes

This marker was evaluated in three studies [20,23] including 265 participants, with 191 children with PH and 104 in the control group. Monocytes were calculated using standard hematologic analyzers, expressed in (1000/µL), and reported as mean ± SD in two studies and as medians and IQR in one study [29]. The pooled result of mean monocytes was significantly higher in the PH group, where MD: 0.08 95CI%: (0.04–0.11), with heterogeneity, where I^2^ = 0% (Figure 4c).

### 3.9. Platelets

**Figure 5:**
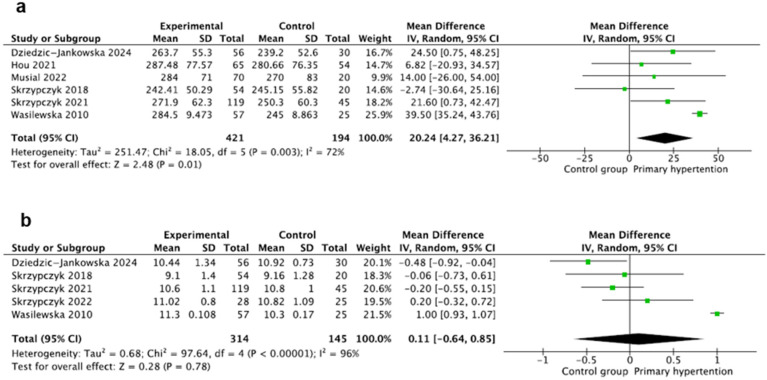
Meta-analysis of mean values of platelets (**a**) and mean platelet volume (MPV) (**b**) [20,23,24,25,26,28,29].

This marker was evaluated in six studies [20,23,24,25,28,29] including 615 participants, with 421 children with PH and 195 children in the control group. Platelet counts were calculated using standard hematologic analyzers and expressed in (1000/µL) and reported as mean ± SD in three studies except for the studies by Wasilewska and Dziedzic-Jankowska, where the authors provided medians and mean and SD values were calculated using the Hozo method [17]. The pooled result of mean platelets was significantly higher in the PH group, where MD: 20.24 95CI%: (4.27–36.21), with significant heterogeneity, where I^2^ = 72% (Figure 5a).

### 3.10. Mean Platelet Volume

This marker was evaluated in five studies [24,25,26,28,29] including 459 participants, with 314 children with PH and 145 healthy children. Mean platelet volume (MPV) was evaluated using standard hematologic analyzers and expressed in all studies as (fL). MPV was reported as mean ± SD except for the study by Wasilewska, where the authors again provided only medians, and mean, and SD values were calculated using the Hozo method [17]. The mean value of MPV was similar in both groups, where MD: 0.11 95%CI: (−0.64–0.85). There was also significant heterogeneity (I^2^ = 96%) (Figure 5b).

### 3.11. Neutrophil-to-Lymphocyte Ratio

**Figure 6:**
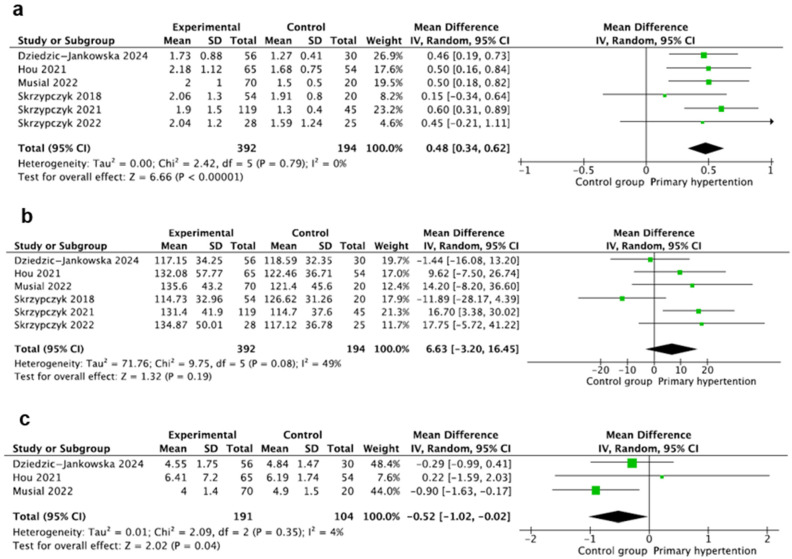
Meta-analysis of mean values of neutrophil-to-lymphocyte (NLR) (**a**), platelet-to-lymphocyte (PLR) (**b**), and lymphocyte-to-monocyte (MLR) ratios (**c**) [20,23,24,25,26,29].

This marker was evaluated in six studies [20,23,24,25,26,29] including 586 participants, with 392 children with PH and 194 in the control group. In all the studies, NLR was calculated as a neutrophil count, i.e., lymphocyte count quotients with the counts evaluated using standard hematologic analyzers and reported as mean ± SD in all studies, except for the study by Dziedzic-Jankowska et al., where the authors reported medians and IQR [29]. The mean value of NLR was significantly increased in the hypertension group compared to controls, where MD: 0.48 95%CI: (0.34–0.62) and I^2^ = 0% (Figure 6a).

### 3.12. Platelet-to-Lymphocyte Ratio

This marker was also evaluated in six studies [20,23,24,25,26,29] including 586 participants, with 392 children with PH and 194 in the control groups. In all the studies, PLR was calculated as a platelet count, i.e., lymphocyte count quotients with the counts evaluated using standard hematologic analyzers and reported as mean ± SD. Mean values of PLR were comparable in both groups, where MD: 6.63 95%CI: (−3.20–16.45). The heterogeneity was moderate, where I^2^ = 49% (Figure 6b).

### 3.13. Lymphocyte-to-Monocyte Ratio

This marker was evaluated in three studies [20,23,29] including 295 participants, with 191 children with PH and 104 in the control groups. In all the studies, LMR was calculated as a lymphocyte count, i.e., monocyte count quotients with the counts evaluated using standard hematologic analyzers and reported as mean ± SD. The mean value of LMR was significantly lower in the PH group compared to controls, where MD: −0.52 95%CI: (−1.02–−0.02) and I^2^ = 4% (Figure 6c).

### 3.14. Additional Markers

The following markers were evaluated in single studies: white blood cell count (WBC), E-selectin, interleukin 18 (IL-18), macrophage inflammatory protein 1α (MIP-1α), MIP-1β, monocyte chemoattractant protein 1 (MCP-1), tumor necrosis factor α (TNFα), regulated on activation, normally T-expressed (RANTES), and angiogenin. WBC (7.65 ± 2.27 vs. 6.70 ± 1.71 (1000/µL); *p* = 0.017) [30], E-selectin (87.4 ± 28 vs. 65.4 ± 22 (ng/mL); *p* = 0.001) [19], MIP-1β (117.9 ± 140.6 vs. 58.3 ± 21.9 (pg/mL); *p* = 0.04) [22], and RANTES (19.7 ± 25.8 vs. 10.7 ± 10.6 (ng/mL); *p* = 0.04) [22] were significantly higher in patients with PH compared to healthy peers without differences in remaining markers.

## 4. Discussion

### 4.1. Summary of Main Findings

To the best of our knowledge, this is the first review summarizing the data on differences in the markers of subclinical inflammation between pediatric patients with primary hypertension and normotensive individuals. We included a satisfactory number of studies to draw reliable conclusions. Our results showed that pediatric patients with PH were characterized by significantly higher low-grade inflammation markers than healthy peers. Hs-CRP and adhesion molecules (ICAM-1 and VCAM-1), as well as simple complete blood count-derived parameters (neutrophil count, monocyte count, platelet count, and neutrophil-to-lymphocyte ratio, were found to be higher, and lymphocyte-to-monocyte ratio lower, in pediatric patients with PH. However, we found no differences in interleukin 6 concentrations, lymphocyte count, PLR, and MPV values. Some of the markers were assessed only in single studies (E-selectin, IL-18, MIP-1β, MIP-1α, MCP-1, TNF-α, angiogenin, and RANTES), and their authors found significant differences between the groups in E-selectin [19], MIP-1β, and RANTES [22].

### 4.2. Comparison with Other Studies

IL-6 is a protein secreted by macrophages in response to specific microbial molecules, referred to as pathogen-associated molecular patterns (PAMPs). IL-6 stimulates the inflammatory processes in many diseases, such as multiple sclerosis, diabetes mellitus, and atherosclerosis [30]. C-reactive protein (CRP) is a protein produced by hepatocytes in response to IL-6. Its physiological role is binding to lysophosphatidylcholine expressed on the surface of apoptotic and necrotic cells and some bacteria (e.g., Streptococcus pneumoniae) to activate the complement system [31]. CRP is a significant cardiovascular risk factor and correlates in adults with atherosclerosis burden and cardiovascular events. The meta-analysis by Jayedi et al. found that higher levels of hs-CRP and IL-6 were associated with the risk of developing hypertension [32]. Our results cannot be translated directly to this study because we could only analyze case–control studies without evaluating patients at different time points. Nevertheless, in our case, hs-CRP concentrations were higher in patients with PH, and we did not show differences for IL-6, but we could only include two studies in the meta-analysis. Based on the results of our meta-analysis and the results of studies in adults, we can conclude that hs-CRP concentration is an important, repeatable biomarker of hypertension and cardiovascular burden in children.

The increase in soluble forms of adhesion molecules, ICAM-1 and VCAM-1, is an indicator of immune system activation and endothelial dysfunction. Their expression on endothelial cells rises in response to different pro-inflammatory stimuli. These molecules facilitate inflammatory cells’ migration into tissues and promote negative sequelae, including atherosclerosis and hypertensive-mediated organ damage. Observational adult studies suggest that the concentration of adhesion molecules, especially ICAM-1, might predict cardiovascular outcome [33,34,35,36]. Similarly, although we evaluated only two studies in our analysis, we found both ICAM-1 and VCAM-1 to be significantly higher in the hypertensive group, making it a promising biomarker for PH in children.

Neutrophils and lymphocytes are crucial components of the immune system. Neutrophils are innate immunity cells that produce cytokines, chemokines, growth factors, and matrix metalloproteinases. Conversely, lymphocytes, which are adaptive immunity cells, control immune response. Neutrophils and lymphocytes interact with each other; thus, their numbers and ratio indicate systemic inflammation [13]. Neutrophils can raise blood pressure by, among other things, generating reactive oxygen species (ROS), increasing endothelial permeability, and inducing vascular dysfunction [37]. On the other hand, a decrease in lymphocyte count is associated with a decline in overall health and stress on the body. The lymphocyte pool represents different subpopulations with different effects on blood pressure. Th17 lymphocytes producing interleukin 17 increase renal sodium reabsorption [38]. Regulatory T lymphocytes lower blood pressure by showing anti-inflammatory effects and producing interleukin 10, which lowers blood pressure and restores endothelial function [39]. A meta-analysis involving twenty adult studies and one pediatric study [24] revealed that hypertensive patients had higher levels of NLR than normotensive individuals [13]. The same results were found in our pediatric data. Our meta-analysis and numerous findings in adults indicate that NLR and even neutrophil count alone may be an inexpensive, widely available additional marker of primary hypertension in developmental age patients.

Monocytes are another part of the innate immune system and play an essential role in the inflammatory reaction. Monocytes migrate from the bloodstream to tissues and differentiate into various immune cells, including dendritic cells, macrophages, and foam cells. This process triggers the secretion of pro-inflammatory cytokines, the production of matrix metalloproteinases, and the formation of reactive oxygen species. This finally leads to a chronic inflammatory response, endothelial cells, and insulin resistance. This eventually results in high blood pressure, diabetes, and obesity. Adult studies have found that reduced LMR is a risk factor for cardiovascular disease [40]. Recently, researchers analyzed data from 4706 patients from the National Health and Nutrition Examination Survey (NHANES) and concluded that increased LMR is independently related to reduced all-cause mortality in patients with obese hypertension [41]. Of the papers we reviewed, monocytes were evaluated in only two studies. We showed that the total number of monocytes was higher and LMR was lower in children with PH. Further and prospective studies are needed to evaluate the usefulness of monocytes/LMR in children with PH.

Higher platelet counts might be both a result and a predisposing factor of inflammatory response. Megakaryocytes are stimulated by inflammatory cytokines and present accelerated proliferation and platelet production. On the other hand, platelets can release thromboxane and other mediators and promote the adhesion and migration of monocytes, which may cause increased inflammation and promote the progression of atherosclerosis [42]. Numerous meta-analyses revealed that elevated PLR was an independent predictor for poor prognosis in adults with both stable and unstable coronary disease [42,43,44,45,46]. Mean platelet volume (MPV), an indicator of platelet size, reflects platelet reactivity. Large platelets have increased aggregability and express more thromboxane and adhesion molecules. Higher MPV values have been found in adult patients with arterial hypertension [47]. Three meta-analyses of adult studies revealed that higher MPV values were associated with increased risk for preeclampsia [48], coronary artery disease [49], and cardiovascular events [50]. Our meta-analysis showed that pediatric patients with PH had higher platelet counts than their healthy peers. Otherwise, no statistically significant differences in PLR and MPV between hypertensive children and children with normal BP were revealed. However, significant heterogeneity between studies should be emphasized.

Other interesting papers on inflammation versus hypertension in children have been published in the last three years. We did not include these papers in our meta-analysis because the authors did not report the exclusion of secondary forms of hypertension in the children studied. Considering that secondary hypertension accounts for at least half of all cases in children [3,51], including these papers could have caused a significant bias. Huang et al., in a study of children with obstructive sleep apnea, showed surprisingly lower levels of TNFα and interleukin 17 in hypertensive patients compared to normotensive children. It is worth noting that in this study, the number of hypertensive children was only ten [52]. The analysis of the HELENA-CSS cohort revealed elevated concentrations of E-selectin, ICAM-1, interleukin 1, and TNFα in hypertensive patients [53]. In the NHANES cohort, hypertensive patients were characterized by higher lymphocyte, neutrophil, monocyte, and platelet counts and higher systemic immune inflammation index (SII), NLR, PLR, and lower LMR [54].

Some studies have also analyzed clinical exponents of subclinical inflammation in association with arterial hypertension. Muñoz Aguilera et al. showed an association between periodontitis, a model of minor inflammation, and the risk of hypertension [55]. Single pediatric papers have shown similar associations between oral health and blood pressure [56,57].

The question is whether therapeutic interventions can lower inflammatory markers. Results regarding different treatment approaches on blood pressure are inconclusive, e.g., in the abovementioned meta-analysis on periodontitis and blood pressure [55]. Nevertheless, a meta-analysis by Takagi et al. revealed that treatment of hypertensive adults with telmisartan significantly reduced IL-6 and TNFα concentrations [58], and a meta-analysis by Soltani et al. showed that adherence to the DASH (Dietary Approaches to Stop Hypertension) diet statistically significantly reduced hs-CRP concentrations in adults [59]. Similarly, two meta-analyses found that non-pharmacological measures might have some impact on ICAM-1 and VCAM-1 concentrations in hypertensive adults [60,61]. Another meta-analysis showed that pentoxifylline use significantly reduced inflammatory markers (hs-CRP and TNFα, but not IL-6), but this did not affect systolic or diastolic blood pressure [62].

### 4.3. Strengths and Limitations

Our literature review involved a comprehensive literature search in the main databases. We implemented the recommendations of the PRISMA guidelines and guidelines from Cochrane Collaboration.

Our review has some limitations. Firstly, we included only case–control studies. All but one of the analyzed studies were performed in Poland. As many as 4/13 studies came from the center of the authors of this manuscript, which may be considered a potential conflict of interest or bias. However, this is how we know the same patients were not analyzed in several papers, and the laboratory methods were consistent. As mentioned in the Materials and Methods, the search strategy was prepared by an independent, recognized expert in the field. In addition, to further reduce the risk of bias, we asked three researchers unrelated to the project to independently review the search strategy, the rejected manuscripts (especially those rejected at the last stage), and the manuscripts included in the study. All experts were in agreement regarding the selection of papers for this meta-analysis.

We could not fully explore the effects of certain markers; this applies especially to IL-6, ICAM-1, and VCAM-1 (two studies for each marker). Moreover, we could not analyze numerous markers as they were evaluated only in single studies (e.g., E-selectin). Almost all studies had sample sizes of less than 100 participants, and the risk of effect overestimation is greater in studies with small sample sizes.

Furthermore, studies used different definitions of arterial hypertension and various protocols to exclude secondary forms of hypertension. Moreover, in most studies, hypertensive patients were characterized by a high proportion of overweight and obese patients, which is typical for primary hypertension in developmental age. There were no differences in body mass index between the PH and control groups in only three studies [18,19,27]. As there is a well-known association between obesity and subclinical inflammation [63], the latter must be considered as a potential bias. Also, hs-CRP, IL-6, and peripheral blood morphology were evaluated using different laboratory techniques.

Finally, there is a possibility that we may have missed publications (e.g., published in the local press in languages other than English). Still, in our opinion, this risk is very small.

### 4.4. Implications for Practice and Further Research

Our results suggest that the implementation of reliable, simple, cheap, repeatable inflammatory markers (i.e., complete blood count, hs-CRP) should be included in the routine evaluation of pediatric patients suspected of arterial hypertension. There is a need for cohort, observational studies examining these markers as predictors for the development of PH. Also, as PH poses a substantial risk for left ventricular hypertrophy and kidney and arterial damage, there is a need for studies evaluating the association between subclinical inflammation and hypertension-mediated organ damage. It is also necessary to assess the impact of therapeutic approaches on inflammatory status in children with PH. Single pediatric data suggest that normalization of blood pressure was associated with a drop in inflammatory markers [64].

## 5. Conclusions

This systematic review and meta-analysis showed that some low-grade inflammation markers were higher in pediatric patients with primary hypertension compared to healthy peers. Concentrations of hs-CRP, adhesion molecules (ICAM-1 and VCAM-1), and complete blood count-derived parameters, such as neutrophil count, monocyte count, platelet count, and neutrophil-to-lymphocyte ratio, were significantly higher in children with primary hypertension, while parameters such as the lymphocyte-to-monocyte ratio were significantly lower. These promising results could lead to validating and employing these markers as future tools for grading hypertension and monitoring antihypertensive treatment. However, the latter requires evaluation in adequately numbered and well-designed studies.

## Figures and Tables

**Table 2:** Risk of bias summary for case–control studies. Adapted from Newcastle–Ottawa scale (retrieved from http://www.ohri.ca/programs/clinical_epidemiology/oxford.asp (accessed on 27 December 2024)).

Quality Assessment Criteria	Criterion to be Fulfilled to Award Asterix (*)	Dziedzic-Jankowska et al. [29]	Gackowska et al. 2020 [14]	Garanty-Bogacka et al.2005 [18]	Głowińska-Olszewska et al. 2007 [19]	Hou et al. 2021 [20]	Kołakowska et al.2018 [21]	Litwin et al.2010 [22]	Musiał et al.2022 [23]	Skrzypczyk et al. 2018 [24]	Skrzypczyk et al.2021 [25]	Skrzypczyk et al.2022 [26]	Trojanek et al.2019 [27]	Wasilewska et al.2010 [28]
Is the case definition adequate?	(a) Yes, with independent validation * (b) Yes, e.g., record linkage or based on self-reports (c) No description	*	*	*	*	*	*	*	*	*	*	*	*	*
Representativeness of the cases	(a) Consecutive or obviously representative series of cases * (b) Potential for selection biases or not stated	*	*	*	*	*	*	*	*	*	*	*	*	*
Selection of controls	(a) Community controls * (b) Hospital controls (c) No description	c	*	c	*	*	b	c	c	c	c	c	*	b
Definition of controls	(a) No history of disease (endpoint) * (b) No description of source	*	*	b	b	*	*	*	*	b	b	b	b	*
Comparability of cases and controls on the basis of the design or analysis	(a) Study controls for presence of primary hypertension* (b) Study controls for body mass index *	*	*	**	**	*	*	*	*	*	*	*	**	*
Ascertainment of exposure	(a) Secure record (e.g., surgical records) * (b) Structured interview blind to case/control status * (c) Interview not blinded to case/control status (d) Written self-report or medical record only (e) No description	*	*	*	*	*	*	*	*	*	*	*	*	*
Same method of ascertainment for cases and controls	(a) Yes * (b) No	*	*	*	*	*	*	*	*	*	*	*	*	*
Non-response rate	(a) Same rate for both groups * (b) Non-respondents described (c) Rate different and no designation	*	*	*	*	*	*	*	*	*	*	*	*	*
	Total score	7	8	7	8	8	7	7	7	6	6	6	8	7

*—the publication meets the criterion with an asterix, **—publication meets the criteria “a” and “b”, b—the publication meets the criterion “b”, c—the publication meets the criterion “c”.

## Data Availability

All data relevant to this review are included in the article or uploaded as Supplementary Information.

## Supplementary Materials

The following supporting information can be downloaded at https://www.mdpi.com/article/10.3390/jcm14072319/s1: Table S1: Search strategy; Table S2: Characteristics of the excluded trials (examples); Table S3: PRISMA 2020 checklist.
