# Supplementary material for: Association of Subclinical Inflammation Markers with Primary Hypertension in Children—A Systematic Review with Meta-Analysis
Source: J Clin Med. 2025 Mar 28;14(7):2319. doi: 10.3390/jcm14072319 (PMC11989401; doi:10.3390/jcm14072319)
Supplement: Supplementary file 1 [file jcm-14-02319-s001.zip › Table S1.pdf]

Table S1. Search strategy

Search number	Query	Results
31	(((((((((((("Child"[Mesh] OR "Adolescent"[Mesh] OR "Pediatrics"[Mesh]) OR (pediatric*[Title/Abstract] OR peadiatric*[Title/Abstract] OR paediatric*[Title/Abstract])) OR (boy[Title/Abstract] OR boys[Title/Abstract] OR boyhood[Title/Abstract])) OR (girl*[Title/Abstract])) OR (kid[Title/Abstract] OR kids[Title/Abstract])) OR (child*[Title/Abstract] OR schoolchild*[Title/Abstract])) OR (adolescen*[Title/Abstract])) OR (juvenil*[Title/Abstract] OR youth*[Title/Abstract])) OR (teen*[Title/Abstract] OR preteen*[Title/Abstract])) OR (underage*[Title/Abstract] OR "under age"[Title/Abstract] OR "under aged"[Title/Abstract])) OR (pubescen*[Title/Abstract])) AND ((((((("Essential Hypertension"[Mesh]) OR ("Hypertension"[Mesh])) OR ("essential hypertension"[Title/Abstract])) OR ("arterial hypertension"[Title/Abstract])) OR ("primary hypertension"[Title/Abstract])) OR ("essential high blood pressure"[Title/Abstract] OR "essential hbp"[Title/Abstract])) OR (((((diastolic[Title/Abstract]) OR (systolic[Title/Abstract])) OR (arterial[Title/Abstract])) OR (blood[Title/Abstract])) AND (pressure[Title/Abstract])))) AND ((((("low grade inflammation"[Title/Abstract] OR "low grade inflammatory"[Title/Abstract]) OR ("subclinical inflammation"[Title/Abstract] OR "subclinical inflammatory"[Title/Abstract])) OR ("inflammatory biomarker"[Title/Abstract] OR "inflammatory biomarkers"[Title/Abstract] OR "inflammatory marker"[Title/Abstract] OR "inflammatory markers"[Title/Abstract])) OR ("C-Reactive Protein"[Mesh])) OR ("high sensitivity c reactive protein"[Title/Abstract] OR "hscrp"[Title/Abstract] OR "hs crp"[Title/Abstract]OR high-sensitivity C-reactive protein[Title/Abstract]))) NOT ((animals[MeSH Terms]) NOT (humans[MeSH Terms]))	**1,103**
30	(((((((((((("Child"[Mesh] OR "Adolescent"[Mesh] OR "Pediatrics"[Mesh]) OR (pediatric*[Title/Abstract] OR peadiatric*[Title/Abstract] OR paediatric*[Title/Abstract])) OR (boy[Title/Abstract] OR boys[Title/Abstract] OR boyhood[Title/Abstract])) OR (girl*[Title/Abstract])) OR (kid[Title/Abstract] OR kids[Title/Abstract])) OR (child*[Title/Abstract] OR schoolchild*[Title/Abstract])) OR (adolescen*[Title/Abstract])) OR (juvenil*[Title/Abstract] OR youth*[Title/Abstract])) OR (teen*[Title/Abstract] OR preteen*[Title/Abstract])) OR (underage*[Title/Abstract] OR "under age"[Title/Abstract] OR "under aged"[Title/Abstract])) OR (pubescen*[Title/Abstract])) AND ((((((("Essential Hypertension"[Mesh]) OR ("Hypertension"[Mesh])) OR ("essential hypertension"[Title/Abstract])) OR ("arterial hypertension"[Title/Abstract])) OR ("primary hypertension"[Title/Abstract])) OR ("essential high blood	1,115

	pressure"[Title/Abstract] OR "essential hbp"[Title/Abstract])) OR (((((diastolic[Title/Abstract]) OR (systolic[Title/Abstract])) OR (arterial[Title/Abstract])) OR (blood[Title/Abstract])) AND (pressure[Title/Abstract])))) AND ((((("low grade inflammation"[Title/Abstract] OR "low grade inflammatory"[Title/Abstract]) OR ("subclinical inflammation"[Title/Abstract] OR "subclinical inflammatory"[Title/Abstract])) OR ("inflammatory biomarker"[Title/Abstract] OR "inflammatory biomarkers"[Title/Abstract] OR "inflammatory marker"[Title/Abstract] OR "inflammatory markers"[Title/Abstract])) OR ("C-Reactive Protein"[Mesh])) OR ("high sensitivity c reactive protein"[Title/Abstract] OR "hscrp"[Title/Abstract] OR "hs crp"[Title/Abstract]OR high-sensitivity C-reactive protein[Title/Abstract]))	
29	(animals[MeSH Terms]) NOT (humans[MeSH Terms])	5,314,482
28	(((("low grade inflammation"[Title/Abstract] OR "low grade inflammatory"[Title/Abstract]) OR ("subclinical inflammation"[Title/Abstract] OR "subclinical inflammatory"[Title/Abstract])) OR ("inflammatory biomarker"[Title/Abstract] OR "inflammatory biomarkers"[Title/Abstract] OR "inflammatory marker"[Title/Abstract] OR "inflammatory markers"[Title/Abstract])) OR ("C-Reactive Protein"[Mesh])) OR ("high sensitivity c reactive protein"[Title/Abstract] OR "hscrp"[Title/Abstract] OR "hs crp"[Title/Abstract]OR high-sensitivity C-reactive protein[Title/Abstract])	114,456
27	"high sensitivity c reactive protein"[Title/Abstract] OR "hscrp"[Title/Abstract] OR "hs crp"[Title/Abstract]OR high-sensitivity C-reactive protein[Title/Abstract]	19,835
26	"C-Reactive Protein"[Mesh]	57,931
25	"inflammatory biomarker"[Title/Abstract] OR "inflammatory biomarkers"[Title/Abstract] OR "inflammatory marker"[Title/Abstract] OR "inflammatory markers"[Title/Abstract]	48,055
24	"subclinical inflammation"[Title/Abstract] OR "subclinical inflammatory"[Title/Abstract]	1,850
23	"low grade inflammation"[Title/Abstract] OR "low grade inflammatory"[Title/Abstract]	8,515
22	(((((("Essential Hypertension"[Mesh]) OR ("Hypertension"[Mesh])) OR ("essential hypertension"[Title/Abstract])) OR ("arterial hypertension"[Title/Abstract])) OR ("primary hypertension"[Title/Abstract])) OR ("essential high blood pressure"[Title/Abstract] OR "essential hbp"[Title/Abstract])) OR (((((diastolic[Title/Abstract]) OR (systolic[Title/Abstract])) OR (arterial[Title/Abstract])) OR (blood[Title/Abstract])) AND (pressure[Title/Abstract]))	736,437
21	((((diastolic[Title/Abstract]) OR (systolic[Title/Abstract])) OR (arterial[Title/Abstract])) OR (blood[Title/Abstract])) AND (pressure[Title/Abstract])	519,425

20	"essential high blood pressure"[Title/Abstract] OR "essential hbp"[Title/Abstract]	14
19	"primary hypertension"[Title/Abstract]	2,520
18	"arterial hypertension"[Title/Abstract]	47,617
17	"essential hypertension"[Title/Abstract]	23,923
16	"Hypertension"[Mesh]	330,570
15	"Essential Hypertension"[Mesh]	2,924
14	((((((((("Child"[Mesh] OR "Adolescent"[Mesh] OR "Pediatrics"[Mesh]) OR (pediatric*[Title/Abstract] OR peadiatric*[Title/Abstract] OR paediatric*[Title/Abstract])) OR (boy[Title/Abstract] OR boys[Title/Abstract] OR boyhood[Title/Abstract])) OR (girl*[Title/Abstract])) OR (kid[Title/Abstract] OR kids[Title/Abstract])) OR (child*[Title/Abstract] OR schoolchild*[Title/Abstract])) OR (adolescen*[Title/Abstract])) OR (juvenil*[Title/Abstract] OR youth*[Title/Abstract])) OR (teen*[Title/Abstract] OR preteen*[Title/Abstract])) OR (underage*[Title/Abstract] OR "under age"[Title/Abstract] OR "under aged"[Title/Abstract])) OR (pubescen*[Title/Abstract])	4,363,420
13	pubescen*[Title/Abstract]	3,127
12	underage*[Title/Abstract] OR "under age"[Title/Abstract] OR "under aged"[Title/Abstract]	7,540
11	teen*[Title/Abstract] OR preteen*[Title/Abstract]	38,954
10	juvenil*[Title/Abstract] OR youth*[Title/Abstract]	218,585
9	adolescen*[Title/Abstract]	414,289
8	child*[Title/Abstract] OR schoolchild*[Title/Abstract]	1,817,422
7	kid[Title/Abstract] OR kids[Title/Abstract]	11,412
6	girl*[Title/Abstract]	185,137
5	boy[Title/Abstract] OR boys[Title/Abstract] OR boyhood[Title/Abstract]	176,294
3	pediatric*[Title/Abstract] OR peadiatric*[Title/Abstract] OR paediatric*[Title/Abstract]	529,856
2	"Child"[Mesh] OR "Adolescent"[Mesh] OR "Pediatrics"[Mesh]	3,544,862
