# Supplementary material for: Association of Subclinical Inflammation Markers with Primary Hypertension in Children—A Systematic Review with Meta-Analysis
Source: J Clin Med. 2025 Mar 28;14(7):2319. doi: 10.3390/jcm14072319 (PMC11989401; doi:10.3390/jcm14072319)
Supplement: Supplementary file 1 [file jcm-14-02319-s001.zip › Table S2.pdf]

Table S2. Characteristics of the excluded trials (examples)

**Author, year**	**Reason for exclusion**
Agostinis-Sobrinho et.al. 2020	Lack of control group
Assadi et.al. 2008	Lack of control group
Bancalari et.al. 2011	Adult population, lack of control group
Becton et.al.2013	Lack of control group
Conkar et.al. 2012	Lack of control group
Cook et.al.2010	Population after surgery procedure
Ford et.al.2003	Lack of control group, study group without hypertension
Galcheva et.al 2011	Chronic disease in study group
Lopez-Jaramillo et.al. 2008	Lack of control group
Ramos-Arellano et.al 2020	Adult population
Sun et al. 2025	Lack of control group
Zhang et al. 2025	No diagnosis for secondary causes of hypertension
